# Smoking cessation in community pharmacy practice–a clinical information needs analysis

**DOI:** 10.1186/2193-1801-2-449

**Published:** 2013-09-11

**Authors:** Maya Saba, Renee Bittoun, Vicky Kritikos, Bandana Saini

**Affiliations:** Faculty of Pharmacy, The University of Sydney, Sydney, NSW 2006 Australia; Brain and Mind Research Institute, The University of Sydney, Sydney, NSW 2006 Australia

**Keywords:** Attitudes, Knowledge, Pharmacy, Questionnaire, Smoking cessation

## Abstract

**Background:**

With the emerging role of pharmacists in implementing smoking cessation services and the recent evidence about smoking cessation pharmacotherapies, a needs analysis to assess baseline knowledge about current smoking cessation practice is needed; hence, training and development in this area can target possible ‘gaps’.

**Objective:**

This study aimed at exploring pharmacy students’ knowledge about and attitudes toward smoking cessation, as compared to practicing community pharmacists and smoking cessation educators. The overall objective was to uncover underlying ‘gaps’ in pharmacy-based smoking cessation practice, particularly clinical gaps.

**Setting:**

Final-year pharmacy students at the University of Sydney, practicing community pharmacists and smoking cessation educators in Australia.

**Method:**

As no previous standard pharmacist-focused smoking cessation knowledge questionnaires exist, a review of the literature informed the development of such a questionnaire. The questionnaire was administered to a cohort of fourth-year pharmacy students at the University of Sydney, practicing pharmacists and smoking cessation educators. Data analysis was performed using Predictive Analytics SoftWare (PASW® Statistics 18). Mean total scores, independent *t*-tests, analysis of variances and exploratory factor analysis were performed.

**Main outcome measure:**

To determine areas of major clinical deficits about current evidence related to smoking cessation interventions at the pharmacy level.

**Results:**

Responses from 250 students, 51 pharmacists and 20 educators were obtained. Smoking educators scored significantly higher than pharmacists and students (*P* < .05), while score differences in the latter two groups were not statistically significant (*P* > .05). All groups scored high on ‘general’ knowledge questions as compared to specialised pharmacologic and pharmacotherapeutic questions. All respondents demonstrated positive attitudes toward the implications of smoking cessation. Factor analysis of the 24-item knowledge section extracted 12 items loading on 5 factors accounting for 53% of the total variance.

**Conclusions:**

The results provide a valid indication of ‘gaps’ in the practice of up-to-date smoking cessation services among Australian pharmacy professionals, particularly in clinical expertise areas involving assessment of nicotine dependence and indications, dosages, adverse effects, contraindications, drug interactions and combinations of available pharmacotherapies. These gaps should be addressed, and the results should inform the design, implementation and evaluation of a pharmacy-based educational training program targeting current clinical issues in smoking cessation.

## Impact of findings on practice

- This study represents the first Australian study assessing knowledge about and attitudes toward smoking cessation among pharmacy students and community pharmacy practitioners.
- It revealed significant clinical/pharmacotherapeutic gaps in the practice of current evidence-based smoking cessation interventions at the pharmacy level.
- It developed and validated a questionnaire to measure and assess smoking cessation-related knowledge and attitudes.
- It provides guidance on key areas for future smoking cessation education/training for pharmacy professionals.

## Introduction

The tobacco epidemic represents one of the biggest public health threats affecting billions of lives (Shafey *et al.,*^2003^). Smoking, as a result, remains to be a *major* cause of preventable mortality and morbidity in the developed world (Chandler and Rennard 2010).

With nicotine dependence being classified as a ‘disease’ in the World Health Organization International Classification of Diseases (ICD-10-CM Diagnosis Codes 2013), addressing individual patient needs and providing adequate treatment requires a thorough understanding of the pharmacology of addiction, specialised therapeutic knowledge and psychosocial intervention skills. Whilst many smokers are able to quit ‘cold turkey’, therapeutic services need to be tailored to meet the demands of recalcitrant smokers who cannot quit unassisted, those who have had previous unsuccessful quit attempts and highly dependent smokers with a greater prospect of relapse (Chapman and MacKenzie 2013). This necessitates that healthcare professionals be equipped with evidence-based knowledge to facilitate smoking cessation interventions.

Smoking cessation research is dynamic, and new revised guidelines suggest different approaches to those used conventionally (Zwar *et al.,*^2012^; National Institute for Health and Clinical Excellence 2008). Current emerging recommendations debunk previous myths regarding the use of smoking cessation aids. For instance, it is now clear that nicotine replacement therapy (NRT) can be used while still smoking, with a view to cutting down as a prelude to quitting (Wennike *et al.,*^2003^; Rennard *et al.,*^2006^). NRT can be used beyond the recommended duration of 8 to12 weeks for as long as needed to help patients quit (Medioni *et al.,*^2005^). In fact, NRT can be continued after smoking lapses to promote recovery of abstinence (Ferguson *et al.,*^2012^). It is also evident that higher doses of NRT are more effective than lower doses, particularly in highly dependent smokers (Stead *et al.,*^2012^). Furthermore, the combination of more than one form of NRT is significantly effective (Stead *et al.,*^2012^). Indeed, NRT exhibits a wide safety profile and can be administered during pregnancy after failure of non-pharmacological interventions, in patients with cardiovascular diseases and in smokers aged 12 years and over (Schroder *et al.,*^2002^; Coleman *et al.,*^2011^; Thomas 2012; Hanson *et al.,*^2003^); though many healthcare professionals would be, based on conventional wisdom, unclear or hesitant to initiate this. Hence again, it is crucial for healthcare practitioners to keep abreast in order to successfully assist smokers in quitting.

Pharmacists, among other primary healthcare professionals, can play a fundamental role in smoking cessation. They represent a highly accessible trained workforce with a wide range of therapeutic expertise. They characterise highly trusted practitioners with whom patients often consult about health and medication-related issues (Pharmacy Guild of Australia 2010). Moreover, in the last 2 decades, community pharmacies have broadened their scope of service to include, besides conventional medicine supply, a variety of specialised services, such as health screening and disease management (Schulz *et al.,*^2001^; Monte *et al.,*^2009^). Pharmacists are well-positioned to provide smoking cessation services within pharmacies, where smoking cessation products are stocked and retailed. They can approach a wide spectrum of patients in need of support, motivation and enhanced awareness about nicotine dependence and avenues of treatment (Li 2010). Additionally, systematic reviews, assessing the effectiveness of pharmacist-delivered smoking cessation interventions, indicated that trained community pharmacists, providing counselling and ongoing support, may have a positive effect on abstinence rates (Roughead *et al.,*^2003^; Sinclair *et al.,*^2004^; Dent *et al.,*^2007^). The counselling delivered by pharmacists ranged in nature from simple advice about the importance of smoking cessation, identifying barriers to quitting and providing motivation to more intensive behavioural approaches such as providing support based on the ‘stage-of-change model’ (Sinclair *et al.,*^2004^). Preliminary studies also suggest that pharmacy-based smoking cessation services are cost-effective (Sinclair *et al.*^1999^; Bauld *et al.,*^2011^).

In Australia, in 2011–12, it was estimated that nearly 20.4% of the adult male and 16.3% of the adult female population were smokers (Australian Bureau of Statistics 2013). These rates are far lower than a quarter of a century ago, and quit attempts have doubled since then (Germain *et al.,*^2012^). In an attempt to provide disincentives to purchase tobacco products, new political and social protocols have recently been instituted countrywide. These included the implementation of the “plain” (no brand images/advertisement) packaging legislation and the raised taxes on cigarettes (Cancer Council Victoria 2012). In Australia, over-the-counter NRT and prescription-only varenicline are available. NRT patches (21 mg/24 hours and 15 mg/16 hours) and varenicline can be accessed through subsidised pricing via the Pharmaceutical Benefits Scheme, for a period of 12 weeks for the patches and up to 24 weeks for varenicline. Despite this infrastructure, little is known about the smoking cessation services provided in this sector. Published data suggest that sales of smoking cessation products have increased, but only a minority of smoking cessation product users report receiving any advice or support (Bittoun 2007; Paul *et al.,*^2003^). Some studies have shown that smokers consistently underutilise NRT, both in terms of the number of pieces administered per day and the duration of time that treatment is used (Ferguson *et al.,*^2011^). For instance, despite Australia’s active pharmacy practice researchers in the areas of asthma, diabetes and Home Medicines Review (Australian Government, Department of Health and Ageing 2009; 2011; The University of Sydney, Faculty of Pharmacy Faculty of Pharmacy 2005), comprehensive pharmacy-delivered smoking cessation programs have not been developed or evaluated yet. This situation may also stem from lack of positive attitudes toward pharmacist-delivered smoking cessation services and the lack of pharmacists’ skills and confidence in being able to provide these services in line with the current therapeutic guidelines. In a previous simulated patient study conducted in Sydney, authors concluded that evidence-based smoking cessation advice in pharmacies was fragile and may be compromised by commercial concerns (Chiang and Chapman 2006). Another unclear issue is whether smoking cessation is handled by pharmacy frontline staff or pharmacists. Anecdotally, it is known that often Australian pharmacy assistants may be in charge of the smoking cessation products area. To add, whilst tobacco education is common in pharmacy schools in the United States (Corelli *et al.,*^2005^), for instance, little is known about the depth and scope of tobacco cessation content in Australian pharmacy curricula. As a result, an identification of needs, preferences and current awareness levels is crucial, as for pharmacists to perform competent smoking cessation interventions throughout their daily practice, they need to attain and demonstrate knowledge, skills and confidence in this field.

Given that early sources of knowledge that contribute to one’s professional career are generally acquired at professional courses, i.e., while undertaking a degree, it was anticipated that mapping the knowledge and attitudes of final-year pharmacy students would be an important marker of the “level of awareness” of pharmacy professionals about smoking cessation. Pharmacy students during their final year serve as a pertinent target population because of their imminent entry into the profession, recency of knowledge acquisition, possible personal smoking status or contact with peers who smoke. It may also be hypothesised that experience with providing smoking cessation services and advice should enhance the skill base of professionals and that practicing professionals would have higher awareness and more positive attitudes toward their role in smoking cessation service provision.

### Aim of the study

Therefore, the aim of this study was to assess the knowledge about and attitudes toward smoking cessation in final-year pharmacy students and to compare the latter with that of practicing pharmacists and specialised smoking cessation educators. Accordingly, the overall objective of this study was to uncover underlying ‘gaps’ in pharmacy-based smoking cessation practice, aiming at investing the findings of this research in providing up-to-date education to improve smoking cessation practice.

## Methods

A cross-sectional questionnaire method was utilised. Since there are no pre-existing standard tools in the literature that assess knowledge and attitudes regarding smoking and smoking cessation, the first step was to design the questionnaire.

### Questionnaire development

**Figure 1:**
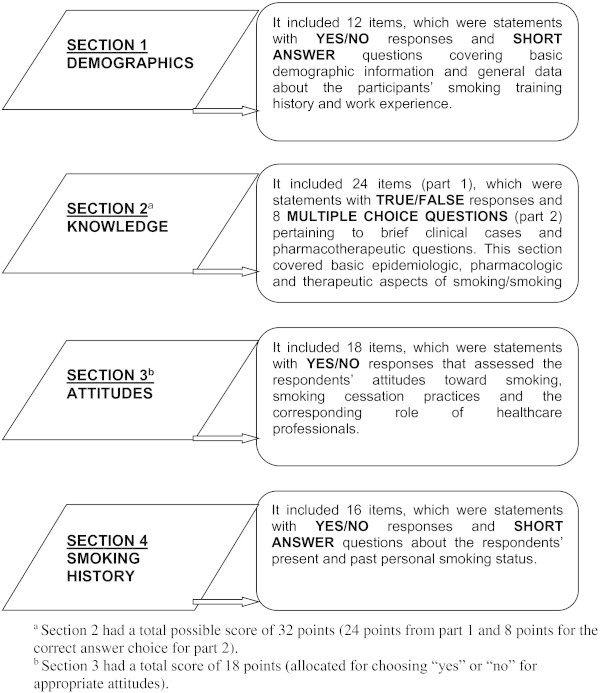
**The SCIP questionnaire.**^a^Section 2 had a total possible score of 32 points (24 points from part 1 and 8 points for the correct answer choice for part 2). ^b^Section 3 had a total score of 18 points (allocated for choosing “yes” or “no” for appropriate attitudes).

The “*Smoking Cessation In Pharmacy* (***SCIP***)” questionnaire was constructed by the principal author based on a review of the literature (Centers for Disease Control and Prevention 2005). The questionnaire comprised 4 sections as highlighted in Figure 1. The face and content validity of the questionnaire were assessed by smoking cessation experts, experienced practicing pharmacists and academic pharmacists specialising in the field of smoking cessation and respiratory health.

### Sample selection

Three discrete groups were selected as the target population–1) pharmacy students, 2) practicing pharmacists and 3) smoking cessation educators. In Australia, the latter consists of a niche group of healthcare professionals, usually specialised respiratory nurses, who have training and experience in supporting patients to quit smoking.

### Sample size

For group 1, a full cohort of pharmacy students, enrolled at one of the largest pharmacy training provider universities in Australia, was selected. There was no existing research on which to base a sample size calculation for groups 2 and 3. We estimated that the knowledge score of pharmacists may be at least 20% higher than that of pharmacy students and that the knowledge score of smoking cessation educators may be at least 40% higher than that of pharmacy students. Then, using a power of 90% and a 2-sided significance level of 5%, a statistical power calculation indicated that the study would require 50 pharmacists and 13 smoking cessation educators. The study was approved by the Human Research Ethics Committee at the University of Sydney, protocol number 13046.

### Recruitment strategies

Group 1- Final year pharmacy students at the University of Sydney, one of the largest pharmacy provider universities in Australia, were recruited. A sampling frame of all fourth-year pharmacy students enrolled in the “Pharmacotherapeutics in Practice” course during semester 2, 2010, at the University of Sydney, was used. Tutors in this course allocated 15 minutes for consenting students to read project information and to complete and hand in the questionnaires within a scheduled tutorial.

Group 2- It was anticipated that pharmacists could be approached via the Pharmaceutical Society of Australia, a national professional pharmacy organisation that represents Australian pharmacists working in various sectors. Accordingly, all pharmacists who attended Continuing Education lectures run by the Pharmaceutical Society of Australia in Sydney during November 2011 were invited to participate. Measures were taken to ensure that none of these lecture topics included smoking cessation-related issues. Questionnaires were handed out prior to the lecture, and consenting pharmacists were requested to hand in the completed questionnaire forms after the completion of the activity.

Group 3- Smoking cessation educators were approached via the Brain and Mind Research Institute Smoking Unit (University of Sydney), which represents a leading Australian body in providing smoking cessation training and conducting smoking cessation-related studies and clinical trials. In November 2011, the questionnaire was emailed to all smoking cessation educators whose names were on the mailing list of the Brain and Mind Research Institute–University of Sydney Smoking Unit network. The inclusion criteria were that participants in this group should have previously completed at least 1 smoking cessation training certificate and were running or working in respiratory/smoking cessation clinics at the time of questionnaire administration. Participants were asked to return the questionnaires upon completion. Questionnaire collection was completed by January 2012.

In all of the 3 groups, the questionnaire was administered along with a participant information statement, which highlighted the objectives of the research and assured participants that their contribution was completely voluntary and strictly confidential. The return of the completed questionnaires was sufficient evidence of consent to participate. No reimbursement for participation in the study was offered.

### Statistical analyses

Data from completed questionnaire was entered into the Predictive Analytics SoftWare package (PASW® Statistics 18) for analysis. Data from the demographic and smoking history sections (SCIP sections 1 and 4) was analysed using descriptive statistics. For Sections 2 and 3, mean total knowledge and attitude scores were calculated. Analysis of variance (ANOVA) and independent sample *t*-tests were utilised to compare results between and within groups. To explore the psychometric properties of the SCIP questionnaire, an exploratory factor analysis was performed for the 24 True/False (T/F) knowledge items (SCIP section 2, part 1). Only this section was included in the factor analysis, as this technique is meaningful only when items are scored the same way. The factor structures were analysed using a principal components analysis and varimax rotation with Kaiser normalization. The Kaiser Criterion (eigenvalues >1), number of steps in the scree plot and the proportion of total variance explained were the criteria implemented for the number of factors to be extracted. Items that had poor factor loadings (<0.30) or that cross-loaded on 2 or more factors were removed from the analysis (Pett *et al.,*^2003^). The reliability of the remaining items was estimated using Cronbach’s alpha coefficient, which measures the homogeneity of items within a scale (Carmines and Zeller 1979).

## Results

A total of 250 students (89.9% response rate), 51 pharmacists (72.9% response rate) and 20 smoking cessation educators (22.2% response rate) completed the questionnaire. Overall, 90.3% of all the participants submitted completed questionnaires, particularly providing answers to all the questions in the knowledge and attitude sections.

Table 1 highlights the general demographic information of respondents. Table 2 presents the mean total knowledge and attitude scores for the 3 participant groups. The mean knowledge score was not significantly different between pharmacists and final-year pharmacy students. However, both groups had significantly lower knowledge scores than smoking cessation educators (*P* < .05). Participants in all 3 groups had positive attitudes toward smoking cessation and its implications.

**Table 1:** **Demographic data of respondents**

Demographics		Pharmacy students	Pharmacists	Smoking educators
**Age**	**20**–**29**	96.8%	51.0%	5.0%
	**30**–**39**	0.8%	11.8%	15.0%
	**40**+	0.0%	29.4%	80.0%
	**Missing data**	2.4%	7.8%	-
**Gender**	**Female**	65.6%	74.5%	75.0%
	**Male**	34.0%	23.5%	25.0%
	**Missing data**	0.4%	2.0%	-
**Tobacco smoking status**	**Smoker**	3.6%	0.0%	0.0%
	**Non**-**smoker**	91.2%	92.2%	100.0%
	**Missing data**	5.2%	7.8%	-
**Smoking training status**	**Yes**	41.6%	80.4%	90.0%
	**No**	58.4%	19.6%	10.0%
	**Missing data**	-	-	-
**Workplace**	**Pharmacy**	81.6%	94.1%	10.0%
	**Other**	17.6%	3.9%	90.0%
	**Missing data**	0.8%	2.0%	-

**Table 2:** **Total knowledge and attitude scores of respondents**

Respondents	Pharmacy students (A)	Pharmacists (B)	Smoking educators (C)	ANOVA post hoc significance
**Mean total knowledge score (%) ± SD**	**63.4% ± 9.8**	**65.2% ± 9.9**	**77.8% ± 6.3**	***A*** **<** ***B*** **<** ***C*:*P*** **< .*001***
	Range:	Range:	Range:	A vs B: P > .05
	34.3–87.5%	37.5–84.4%	65.6–90.6%	*A vs C*: *P* < .*05*
				*B vsC*: *P* < .*05*
Mean score–General knowledge section (SCIP section 2, part 1–24 T/F)	71.4% ± 10.5	72.3% ± 10.5	85% ± 6.5	***A*** **<** ***B*** **<** ***C*:*P*** **< .*001***
				A vs B: P > .05
				*A vs C*: *P* < .*05*
				*B vs C*: *P* < .*05*
Mean Score–Clinical/therapeutic section (SCIP section 2, part 2–8 MCQs)	39.4% ± 18.0	43.9% ± 17.4	56.3% ±15.4	***A*** **<** ***B*** **<** ***C*:*P*** **< .*001***
				A vs B: P > .05
				*A vs C*: *P* < .*05*
				*B vs C*: *P* < .*05*
**Mean total attitude score (%) ± SD**	**88**.**8%** ± **10**.**7**	**88**.**7%** ± **8**.**17**	**92**.**5%** ± **5**.**8**	**A** < **B** < **C**: **P** = .**279**
	Range:	Range:	Range:	A vs B: P > .05
	33.3–100%	66.7–100%	83.3–100%	A vs C: P > .05
				B vs C: P > .05

Significant *P* values are italicised.

The 32 knowledge questions in Section 2 of the questionnaire were then categorised into separate themes, for which total scores were also calculated and compared among the groups, as shown in Table 3. It was interesting to note that when asked about the adverse effects of smoking or the constituents of a cigarette, the scores were much higher in all groups than when participants were asked about specific pharmacologic- or pharmacotherapeutic-related issues. Accordingly, the proportions of correct answers for the clinical/pharmacotherapeutic knowledge section (SCIP section 2, part 2) were compared in Table 4. Both tables indicate that ‘general’ knowledge was higher in all 3 groups as compared with more specific professional knowledge. SCIP section 2 part 1 comprised the ‘general knowledge’ section that included a variety of questions related to smoking and smoking cessation, such as the prevalence of smoking in Australia, the adverse effects of smoking, nicotine dependence and general pharmacotherapeutic-related issues acquired throughout daily practice. On the other hand, SCIP section 2 part 2 consisted of more specialised pharmacotherapeutic questions and clinical case scenarios. It was anticipated that practitioners, who have previously received smoking cessation training or those with extensive knowledge and expertise in the field of smoking cessation, would respond to this section better than others. Appendix 1 and Appendix 2 present both parts of the SCIP questionnaire knowledge section.

**Table 3:** **Total scores of knowledge questions** (**SCIP Section2**) **classified into specific smoking**-**related themes**

	Total knowledge score (%)			
Knowledge section (32 items) categories	Pharmacy students (A)	Pharmacists (B)	Smoking educators (C)	ANOVA post hoc significance
**Epidemiology (3Qs)**	54% ± 24.5	54.3% ± 29	81.7% ± 22.9	***A*** < ***B*** < ***C***: ***P*** < .***001***
				A vs B: P > .05
				*A vs C*: *P* < .*05*
				*B vs C*: *P* < .*05*
**Adverse effects of smoking (3Qs)**	91.7% ± 16	92% ± 14.3	90% ± 15.7	**A** < **B** < **C**: **P** = .**873**
				A vs B: P > .05
				A vs C: P > .05
				B vs C: P > .05
**Forms of tobacco smoking (2Qs)**	86% ± 26.9	85.5% ± 27	100%	**A** < **B** < **C**: **P** = .**068**
				A vs B: P > .05
				*A vs C*: *P* < .*05*
				*B vs C*: *P* < .*05*
**Cigarette constituents(2Qs)**	91% ± 20.3	85.5% ± 23	87.5% ± 22.2	**A** < **B** < **C**: **P** = .**180**
				A vs B: P > .05
				A vs C: P > .05
				B vs C: P > .05
**Pharmacology of dependence (4Qs)**	75.3% ± 22.6	80.5% ± 18.3	87.5% ± 17.2	***A*** < ***B*** < ***C***: ***P*** = .***025***
				A vs B: P > .05
				*A vs C*: *P* < .*05*
				B vs C: P > .05
**Assessment of Dependence (3Qs)**	43% ± 27.6	43% ± 26.1	56.7% ± 21.9	**A** < **B** < **C**:**P** = .**092**
				A vs B: P > .05
				*A vs C*: *P* < .*05*
				*B vs C*: *P* < .*05*
**Pharmacotherapy (15Qs)**	53.8% ± 14.1	57% ± 15.9	72% ± 10.9	***A*** < ***B*** < ***C***: ***P*** < .***001***
				A vs B: P > .05
				*A vs C*: *P* < .*05*
				*B vs C*: *P* < .*05*

Significant *P* values are italicised.

**Table 4:** **Proportion of correct answers for clinical**/**pharmacotherapeutic knowledge items (SCIP section 2, part 2)**

	Proportion of correct answers (%)		
Overview of SCIP knowledge section 2 part 2 questions (8 MCQs)	Pharmacy students	Pharmacists	Smoking educators
Quitting during pregnancy	64.8%	80.4%	100%
Smoking cessation in COPD with a history of major depression	61.2%	66.7%	80%
Contraindication of smoking cessation aids	20.8%	25.5%	15%
Age-related benefits of quitting (Fletcher-Peto graph)	56.4%	58.8%	70%
Tobacco-drug interactions	43.2%	39.2%	70%
Dosage of NRT (Possible reason for failure)	48.4%	60.8%	95%
Side effects of smoking cessation aids	5.2%	11.8%	20%
Smoking cessation in cardiovascular conditions	14.8%	7.8%	20%

**Figure 2:**
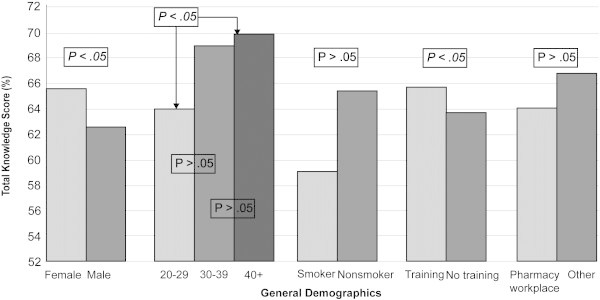
**Impact of various respondent**-**related criteria on total knowledge score.**

**Figure 3:**
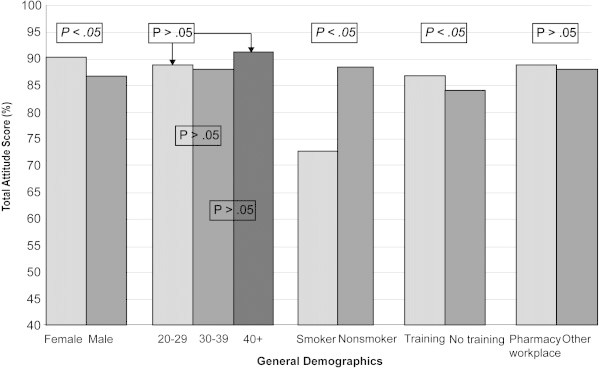
**Impact of various respondent**-**related criteria on total attitude score.**

Figures 2 and 3 illustrate total knowledge and attitude score variability with respect to participant demographics. Table 5 summarises the ANOVA results for the latter. It was interesting to note that the recipients of some form of training in smoking cessation had significantly higher knowledge and more positive attitudes as compared with those without this training.

**Table 5:** **ANOVA results for the factors predicting knowledge and attitude scores**

	Scores between groups	F	Significance ( ***P*** -value)
**Gender**	Total knowledge	3.151	.*044*
	Total attitude	3.738	.*025*
**Age**	Total knowledge	3.036	.*018*
	Total attitude	.394	.813
**Smoking status**	Total knowledge	10.435	.*000*
	Total attitude	15.484	.*000*
**Smoking training history within university settings**	Total knowledge	1.277	.280
	Total attitude	0.773	.462
**Smoking training history outside university settings**	Total knowledge	2.928	.055
	Total attitude	3.918	.*021*
**Pharmacy**-**based workplace**	Total knowledge	1.810	.165
	Total attitude	0.461	.631

Significant *P* values are italicised.

For the 24-item knowledge section, factor analysis extracted 5 primary factors with eigenvalues greater than unity, accounting for 53% of the total variance and a Kaiser–Meyer–Olkin measure of sampling adequacy of 0.6. Twelve items from the questionnaire were removed because of poor factor loading or cross loading on 2 factors. Thus, a 12-item version of the knowledge section was retained after factor analysis and is referred to as the SCIPV2 (Smoking Cessation In Pharmacy questionnaire Version 2). The 12 remaining items covered prevalence, pharmacology, assessment of dependence and pharmacotherapeutic issues. Table 6 summarises the factor loadings and variance contribution of the items retained in the knowledge section of SCIPV2. A reliability analysis of the 12 retained items returned a Cronbach’s alpha coefficient of 0.4. The total knowledge scores were then recalculated for the 12 retained items and compared among groups (Table 7). The pattern was similar to that found in the original SCIP questionnaire, i.e., the mean total knowledge score for the 12-item version was not significantly different between pharmacists and pharmacy undergraduate students. However, both groups had significantly lower knowledge scores than smoking cessation educators (*P* < .05). Table 8 summarises the various smoking cessation elements that need to be addressed in future research.

**Table 6:** **Principal component estimates of the varimax factor loadings for the 24 T**/**F knowledge items of the questionnaire**

Factors	Factor loading	Total variance
***Factor 1***–***Common myths of smoking***		13.8%
Item 7	0.584	
Item 8	0.524	
Item 17	0.631	
Item 19	0.500	
***Factor 2***–***Nicotine replacement therapy knowledge***		11.5%
Item 20	0.767	
Item 21	0.800	
***Factor 3***–***Specialised smoking knowledge***		9.9%
Item 6	0.742	
Item 24	0.701	
***Factor 4***–***General smoking knowledge***		9.0%
Item 1	0.778	
Item 18	0.486	
***Factor 5***–***Nicotine dependence***		8.8%
Item 15	0.793	
Item 23	0.599

**Table 7:** **Total knowledge scores of the 12 items extracted by factor analysis**

Respondents	Pharmacy students (A)	Pharmacists (B)	Smoking educators (C)	ANOVA post hoc significance
**Mean score–general knowledge section (Factor analysis 12 items)**	**62**.**9%** ± **15**.**7**	**63**.**4%** ± **17**.**5**	**82**.**9%** ± **10**.**6**	*A* < *B* < *C*: *P* < .*001*
	Range:	Range:	Range:	A vs B: P > .05
	16.6–91.6%	25–91.7%	58.3–100%	*A vs C*: *P* < .*05*
**Mean TOTAL knowledge score (%) ± SD (12 items + 8 MCQs)**	**53**.**5%** ± **13**.**1**	**55**.**6%** ± **14**.**1**	**72**.**3%** ± **9**.**5**	*A* < *B* < *C*: *P* < .*001*
	Range:	Range:	Range:	A vs B: P > .05
	20–85%	20–85%	55–85%	*A vs C*: *P* < .*05*
				*B vs C*: *P* < .*05*

Significant *P* values are italicised.

**Table 8:** **Smoking cessation elements to be addressed in future research**

1	Overview of smoking–prevalence, adverse effects, benefits of quitting
2	Nicotine dependence–the ‘disease’
3	Pharmacokinetics of nicotine
4	Pathophysiology of dependence
5	Assessment of nicotine dependence–Fagreström test, expired CO levels, urine/salive cotinine
6	Smoking cessation counselling–behavioural interventions, advice, support, lifestyle modifications
7	Smoking cessation pharmacotherapy–products, dosage forms, dosages, instructions for use, duration of therapy, adverse effects, precautions, contraindications, interactions
8	Debunking myths–Safety of NRT, combinations of NRT, concomitant smoking and NRT use, cut-down smoking regimens

## Discussion

Currently, there is no published literature assessing Australian community pharmacists’ awareness about and attitudes toward smoking-related health issues or smoking cessation (El Hajj *et al.,*^2012^; Saulle *et al.,*^2013^; Vanderhoek *et al.,*^2013^; Moxham *et al.,*^2012^); nor are there any valid instruments measuring these factors. Given the new clinical guidelines for the use of various pharmacotherapies (Wennike *et al.,*^2003^), it is imperative that smoking cessation-related knowledge be updated for practicing pharmacists. This study has identified clear ‘gaps’ in knowledge levels among Australian pharmacy professionals, particularly in clinical expertise areas involving assessment of nicotine dependence and indications, dosages, adverse effects, contraindications, drug interactions and combinations of available pharmacotherapies (Beard *et al.,*^2012^). Further, a smoking cessation questionnaire was developed and psychometrically evaluated with satisfactory results. This instrument can be used in other studies and as a basis for the evaluation of educational interventions that focus on smoking cessation.

It was expected that smoking cessation educators, who work with smokers on a daily basis, would achieve the highest knowledge and attitude scores, and the obtained results confirmed this assumption. Practicing community pharmacists, who also encounter patients who smoke or those requesting assistance with smoking cessation, were expected to exhibit more positive attitudes and superior knowledge levels as compared to pharmacy students. However, the results indicated no statistically significant difference between the 2 groups. It should be noted, though, that 51% of the pharmacists who completed the questionnaire were in the 20–29 age group. This indicates that nearly half of the respondents in group 2 were fresh pharmacy graduates with minimal practical experience. This may have contributed to the absence of statistical difference in total scores between pharmacists and pharmacy students. Results indicated that recipients of some form of training demonstrated better knowledge (Zhang *et al.,*^2012^). Non-smokers were also expected to score higher than smokers (Yang *et al.,*^2010^); nevertheless, results revealed that, although non-smokers exhibit more positive attitudes, both groups share similar levels of knowledge. Gender was an unexpected contributor to the variance in knowledge, with females exhibiting higher knowledge levels and more positive attitudes than males.

Besides having positive attitudes toward smoking cessation and the corresponding role of health professionals, interestingly, all 3 groups exhibited a certain base level of ‘general’ knowledge about smoking and smoking health-related issues. For instance, when asked whether nicotine is the most addictive substance in a cigarette or whether passive smoking is harmful to the health, a high proportion of participants in all groups responded correctly. This basic knowledge could be attributed to the fact that smoking has become a common public health issue and is a major focus of various public health campaigns and mass media messages. On the other hand, questions related to prevalence of smoking, pharmacology of nicotine dependence, methods of assessment of nicotine dependence and pharmacotherapy generated significantly lower scores. For items testing knowledge of specific pharmacotherapeutic issues including dosages, side effects, interactions and contraindications of smoking cessation aids, smoking cessation educators scored lower than pharmacists. For instance, when asked which smoking cessation therapy is contraindicated in patients with severe hepatic impairment, only 16% of smoking cessation educators answered correctly as compared with 27% of pharmacists and 23% of students. However, when asked about the therapy that could be associated with the development of suicidal ideations, only 6% of students, 13% of pharmacists and 20% of educators answered correctly. This could be explained by the fact that little is known about smoking cessation aids beyond what is provided on the pack by the manufacturers of NRT. Overall, the mean knowledge scores for the various smoking-related themes matched the total scores obtained by the 3 groups for both the general and clinical-based knowledge sections; thus, this trend indicates a reasonable extraction process and construct validity.

The fact that pharmacists’ level of awareness is not greater than that of students is a matter of concern. In a previous simulated patient study conducted in Australia, authors concluded that evidence-based smoking cessation advice in pharmacies was sub-optimal (Chiang and Chapman 2006).

Factor analysis was used to establish construct validity of the questionnaire. Although the reliability estimate was low as characterised by the obtained Cronbach’s alpha coefficient, this may be accounted for by the fact that smoking and smoking cessation are viewed as general social and public health topics, and therefore knowledge levels in such areas are expected to be diverse. Total scores for the SCIPV2 (12 retained T/F items of knowledge section), were significantly lower than the scores for the 24-item original version, thus confirming that the factor analysis exercise allowed the deletion of superfluous items, leaving a more streamlined version. Score patterns obtained by the respective groups (students = pharmacists < experts) were similar before and after factor analysis, i.e., on both versions of the SCIP, which indicates consistency.

A possible limitation of this study could be the small sample size of smoking cessation educators (n = 20) as compared with pharmacists (n = 51) and students (n = 250). Furthermore, the methods of sample recruitment used for groups 2 and 3 may have led to selection bias, as people with strong beliefs or substantial knowledge, or those interested in continuing education may be more willing to respond to the questionnaire than others.

Future directions should explore the development, implementation and evaluation of pharmacist-directed smoking cessation educational programs that focus mainly on ‘deficit’ areas. Such programs should, therefore, incorporate diverse topics, including pharmacokinetics of nicotine and pharmacology of dependence essential for dictating pharmacotherapy (Benowitz 2008), different methods for assessing dependence, such as carbon monoxide (CO) monitoring and utilising the Fagerström test, psychosocial behavioural interventions and a thorough exposure to various pharmacotherapeutic aspects in light of current clinical guidelines, as highlighted in Table 8. Tobacco cessation education should all also be introduced into the academic curricula of pharmacy and medical students.

## Conclusions

Overall results of this study indicate the presence of major clinical deficits about current evidence related to smoking cessation interventions at the pharmacy level. This study not only developed and validated a questionnaire to measure and assess smoking cessation-related knowledge, but also provides guidance on key areas for future smoking cessation education/training for pharmacy professionals. Given the evidence for pharmacist-delivered smoking cessation interventions and consumer acceptance of the pharmacy as a venue for smoking cessation services (Patwardhan and Chewning 2010), the time is now pertinent to disseminate research on the effect of structured smoking cessation educational training programs on pharmacy students’ and pharmacists’ awareness and confidence in the practice of smoking cessation services.

## Appendix 1

### SCIP knowledge section (Section 2) part 1 with a True/False response and the 12-item questions of SCIPV2 yielded by factor analysis in bold

1. In the last decade, smoking rates in Australia have increased significantly. (F)
2. Besides the pulmonary adverse effects, smoking causes cancer in various organs such as the bladder, intestines and cervix. (T)
3. Deaths attributable to tobacco use in Australia outnumber those caused by AIDS, legal drugs, illegal drugs, road accidents, murder and suicide combined. (T)
4. Passive smoking is not harmful to health. (F)
5. In smoking mothers, neonatal death may occur secondary to tobacco smoke exposure. (T)
6. **People with respiratory diseases, such as asthma, tend to smoke more. (T)**
7. **Rolling your own cigarettes is safer than the packaged industrial brands**. **(F)**
8. **The hubble**- **bubble which uses the water mechanism is a safer way to smoke nicotine. (F)**
9. Nicotine is the most addictive substance in a cigarette. (T)
10. Nicotine is the most harmful substance in a cigarette. (F)
11. In clinical settings, measuring CO levels is one of the most reliable tests of nicotine dependence. (T)
12. Nicotine dependence is a chronic relapsing disease. (T)
13. Nicotine dependence is mediated by dopamine within the reward system of the brain. (T)
14. Nicotine withdrawal symptoms are associated with increased noradrenergic outflow, secondary to deactivation of the reward system. (T)
15. **A heavy smoker is defined as someone who smokes a total of 15 or more cigarettes**/**day**. **(F)**
16. In Australia, smokers can be referred to specialised smoking cessation clinics for treatment. (T)
17. **Younger smokers find it easier to quit than older smokers who have been smoking for a longer time**. **(F)**
18. **The combination of behavioural and pharmacological therapy has been shown to be as effective as each alone**. **(F)**
19. **Nicotine replacement sublingual tablets and patches are more effective than gums**, **lozenges and inhalers**. **(F)**
20. **One should never smoke if he**/**she is on nicotine replacement therapy (NRT). (F)**
21. **Combining different forms of NRTs is contraindicated**. **(F)**
22. Varenicline is considered safe in smokers younger than 18 years. (F)
23. **Relapse is uncommon if patients comply with their optimal smoking cessation plan**. **(F)**
24. Some anti-depressants and anti-hypertensives can be used as smoking cessation therapeutic options**. (T)**

## Appendix 2

### SCIP knowledge section (Section 2) part 2 with correct answers in bold

1. M.N. is a 25 year old pregnant female who is trying to quit smoking. Which statement is the most applicable?
  A. Trying to quit smoking during pregnancy is unsafe due to the development of withdrawal symptoms in the baby.
  B. Check her smoking status first. If she is not highly dependent, she may not need to quit.
  C. Behavioural therapy is not recommended during pregnancy.
  D. **NRT is safe during pregnancy and can be used effectively**.
  E. Champix is the most effective treatment to be used during pregnancy.
2. D.B. is a 50 year old female suffering from severe hypertension and major depression, associated with several unsuccessful suicide attempts. She has been smoking 20 cigarettes/day for 25 years and was recently diagnosed with COPD. Her current medications include fluoxetine, diltiazem and perindopril. Which agent would you recommend to help D.B. quit smoking?
  A. **NRT**
  B. Bupropion
  C. Clonidine
  D. Champix
  E. Combination NRT and Bupropion
3. Which of the following is contraindicated in patients with severe renal and hepatic impairment?
  A. NRTs
  B. Bupropion
  C. Varenicline
  D. **None of the above**
  E. All of the above
4. J.A. is a 60 year old heavy smoker. His grandchildren are refusing to sit next to him because “poppy stinks”. Which of the following statements are correct?
  A. Stopping smoking at the age of 60 will not restore any of his lung function.
  B. If he had quit smoking at the age of 45, his lung function would have improved and become similar to that of a non-smoker.
  C. **If he quits now**, **his lung function will improve by up to 10% within 9 months**.
  D. Quitting smoking now will not decrease his risk of developing a heart attack.
  E. Quitting smoking now will not reduce his risk of developing lung cancer.
5. Tobacco smoke interacts with which of the following:
  A. Insulin
  B. Warfarin
  C. Clozapine
  D. None of the above
  E. **All of the above**
6. E.Y. is a 35 year old male who smokes 25 cigarettes/day and is trying to quit smoking. He has been counselled by his pharmacist to start Step 1 of Nicorette® 15 mg patch as follows: For 8 weeks, apply 1 patch every morning, and remove it before going to bed. Eight weeks later, E.Y. claims that he has completely followed the instructions yet was unsuccessful; therefore he wants to change his current treatment. What could be the most possible reason behind E.Y.’s failure to quit?
  A. He has no will power to quit.
  B. He must have been smoking while he was on the patch.
  C. Step 1 of a 16 hour patch should have been started rather than that of a 24 hour patch.
  D. **One 15 mg patch per day is probably too low a dose in his case.**
  E. None of the above
7. The development of suicidal ideations is a common side effect of smoking cessation therapy for patients on:
  A. NRTs
  B. Bupropion
  C. **Champix**
  D. None of the above
  E. All of the above
8. A.K. is a 55 year old male who has been a chronic smoker since 20 years of age. 12 hours ago, he had a myocardial infarction. Being hospitalised, he was unable to smoke, and he is starting to feel restless. His doctor decided to start a smoking cessation plan. Which therapy will be most appropriate to start A.K. on, 12 hours post his infarction?
  A. Nicotine patch
  B. Nicotine gum
  C. Bupropion
  D. Champix
  E. **None of the above**

## Abbreviations

SCIP: Smoking cessation In pharmacy
NRT: Nicotine replacement therapy
CO: Carbon monoxide
SCIPV2: Smoking cessation In pharmacy questionnaire version 2.

## References

[CR1] (2013). Smoking.

[CR2] (2009). Asthma Pilot Program-Fact Sheet.

[CR3] (2011). Medication Management Reviews.

[CR4] Bauld L, Boyd KA, Briggs AH, Chesterman J, Ferguson J, Judge K, Hiscock R (2011). One-year outcomes and a cost-effectiveness analysis for smokers accessing group-based and pharmacy-led cessation services. Nicotine Tob Res.

[CR5] Beard E, McDermott M, McEwen A, West R (2012). Beliefs of stop smoking practitioners in United Kingdom on the use of nicotine replacement therapy for smoking reduction. Nicotine Tob Res.

[CR6] Benowitz NL (2008). Clinical pharmacology of nicotine: implications for understanding, preventing, and treating tobacco addiction. Clin Pharmacol Ther.

[CR7] Bittoun R (2007). A decade of over the counter therapeutic nicotine in Australia: Its contribution to improving quit rates and saving lives.

[CR8] (2012). Smokefree generation within reach after high court upholds plain packaging.

[CR9] Carmines EG, Zeller RA (1979). Reliability and validity assessment.

[CR10] Centers for Disease Control and Prevention (2005). Tobacco use and cessation counseling—global health professionals survey pilot study, 10 countries, 2005. MMWR Morb Mortal Wkly Rep.

[CR11] Chandler MA, Rennard SI (2010). Smoking cessation. Chest.

[CR12] Chapman S, MacKenzie R (2013). There’s nothing that succeeds like failure: discerning the woods from the trees in smoking cessation debates. Nicotine Tob Res.

[CR13] Chiang P, Chapman S (2006). Do pharmacy staff recommend evidenced-based smoking cessation products? A pseudo patron study. J Clin Pharm Ther.

[CR14] Coleman T, Chamberlain C, Cooper S, Leonardi-Bee J (2011). Efficacy and safety of nicotine replacement therapy for smoking cessation in pregnancy: systematic review and metaanalysis. Addiction.

[CR15] Corelli RL, Kroon LA, Chung EP, Sakamoto LM, Gundersen B, Fenlon CM, Hudmon KS (2005). Statewide evaluation of a tobacco cessation curriculum for pharmacy students. Prev Med.

[CR16] Dent LA, Harris KJ, Noonan CW (2007). Tobacco interventions delivered by pharmacists: a summary and systematic review. Pharmacotherapy.

[CR17] El Hajj MS, Al Nakeeb RR, Al-Qudah RA (2012). Smoking cessation counseling in Qatar: community pharmacists’ attitudes, role perceptions and practices. Int J Clin Pharm.

[CR18] Ferguson SG, Shiffman S, Gitchell JG (2011). Nicotine replacement therapies: patient safety and persistence. Patient Relat Outcome Meas.

[CR19] Ferguson SG, Gitchell JG, Shiffman S (2012). Continuing to wear nicotine patches after smoking lapses promotes recovery of abstinence. Addiction.

[CR20] Germain D, Durkin S, Scollo M, Wakefield M (2012). The long-term decline of adult tobacco use in Victoria: changes in smoking initiation and quitting over a quarter of a century of tobacco control. Aust N Z J Public Health.

[CR21] Hanson K, Allen S, Jensen S, Hatsukami D (2003). Treatment of adolescent smokers with the nicotine patch. Nicotine Tob Res.

[CR22] (2013). Nicotine dependence F17. 2013 ICD-10-CM Coding Reference.

[CR23] Li R (2010). Smoking cessation. British Columbia Drug and Poison Information Centre.

[CR24] Medioni J, Berlin I, Mallet A (2005). Increased risk of relapse after stopping nicotine replacement therapies: a mathematical modelling approach. Addiction.

[CR25] Monte SV, Slazak EM, Albanese NP, Adelman M, Rao G, Paladino JA (2009). Clinical and economic impact of a diabetes clinical pharmacy service program in a university and primary care-based collaboration model. J Am Pharm Assoc.

[CR26] Moxham L, Dwyer T, Reid-Searl K (2012). Graduate nurses and nursing student’s behaviour: knowledge and attitudes toward smoking cessation. Nurse Educ Today.

[CR27] (2008). Smoking cessation services.

[CR28] Patwardhan PD, Chewning BA (2010). Tobacco users’ perceptions of a brief tobacco cessation intervention in community pharmacies. J Am Pharm Assoc.

[CR29] Paul CL, Walsh RA, Girgis A (2003). Nicotine replacement therapy products over the counter: real-life use in the Australian community. Aust N Z J Public Health.

[CR30] Pett MA, Lackey NR, Sullivan JJ (2003). Making sense of factor analysis: the use of factor analysis for instrument development in health care research.

[CR31] (2010). The roadmap: the strategic direction for community pharmacy.

[CR32] Rennard SI, Muramoto M, Glover E, Danielsson T, Landfeldt B, Westin A, Franzon M, Sawe U (2006). Efficacy of nicotine inhaler in smoking reduction: a double blind randomized trial. Nicotine Tob Res.

[CR33] Roughead L, Semple S, Vitry A (2003). The value of pharmacist professional services in the community setting: a systematic review of the literature 1990–2002. Quality Use of Medicines and Pharmacy Research Centre.

[CR34] Saulle R, Bontempi C, Baldo V, Boccia G, Bonaccorsi G, Brusaferro S (2013). GHPSS multicenter Italian survey: smoking prevalence, knowledge and attitudes, and tobacco cessation training among third-year medical students. Tumori.

[CR35] Schroder DR, Ogburn PL, Hurt RD, Crogham IT, Ramin KD, Offord KP, Moyer TP (2002). Nicotine patch use in pregnant smokers: smoking abstinence and delivery outcomes. J Matern Fetal Neonatal Med.

[CR36] Schulz M, Verheyen F, Mühlig S, Müller JM, Mühlbauer K, Knop-Schneickert E (2001). Pharmaceutical care services for asthma patients: a controlled intervention study. J Clin Pharmacol.

[CR37] Shafey O, Dolwick S, Guindon GE (2003). Tobacco control country profiles.

[CR38] Sinclair HK, Silcock J, Bond CM, Lennox AS, Winfield AJ (1999). The cost-effectiveness of intensive pharmaceutical intervention in assisting people to stop smoking. Int J Pharm Pract.

[CR39] Sinclair HK, Bond CM, Stead LF (2004). Community pharmacy personnel interventions for smoking cessation. Cochrane Database Syst Rev.

[CR40] Stead LF, Perera R, Bullen C, Mant D, Hartmann-Boyce J, Cahill K (2012). Nicotine replacement therapy for smoking cessation. Cochrane Database Syst Rev.

[CR41] (2005). Pharmacy Diabetes Care Program.

[CR42] Thomas D (2012). Smoking and cardiovascular diseases. Rev Prat.

[CR43] Vanderhoek AJ, Hammal F, Chappell A, Wild TC, Raupach T, Finegan BA (2013). Future physicians and tobacco: an online survey of the habits, beliefs and knowledge base of medical students at a Canadian university. Tob Induc Dis.

[CR44] Wennike P, Danielsson T, Landfeldt B, Westin A, Tonnesen P (2003). Smoking reduction promotes smoking cessation: results from a double blind, randomized, placebo controlled trial of nicotine gum with 2 year follow-up. Addiction.

[CR45] Yang J, Hammond D, Driezen P, Fong GT, Jiang Y (2010). Health knowledge and perception of risks among Chinese smokers and non-smokers: findings from the Wave 1 ITC China Survey. Tob Control.

[CR46] Zhang CM, Xiao D, West R, Michie S, Troughton R, Hajek P (2012). Evaluation of 3-day smoking cessation training course for doctors from 38 cities in China. Chin Med J (Engl).

[CR47] Zwar N, Richmond R, Borland R, Peters M, Litt J, Bell J (2012). Supporting smoking cessation: a guide for health professionals.

